# Long-term Bowel function and pediatric health-related quality of life after transanal rectal mucosectomy and partial internal anal sphincterectomy pull-through for Hirschsprung Disease

**DOI:** 10.3389/fped.2023.1099606

**Published:** 2023-02-14

**Authors:** Zhen Zhang, Qi Li, Bo Li, Mashriq Alganabi, Long Li

**Affiliations:** ^1^ Department of General Surgery, Capital Institute of Pediatrics, Beijing, China; ^2^ Translational Medicine Program, Division of General and Thoracic Surgery, The Hospital for Sick Children, Toronto, ON, Canada

**Keywords:** Hirshsprung’s disease, aganglionosis, TRM-PIAS, bowel function, HAEC

## Abstract

**Objective:**

The aim of this study was to define controlled outcomes for bowel function and quality of life (QoL) after transanal rectal mucosectomy and partial internal anal sphincterectomy pull-through (TRM-PIAS, A modified Swenson procedure) for Hirschsprung disease (HD).

**Background:**

We have previously shown that a novel modification of transanal rectal mucosectomy and partial internal anal sphincterectomy (TRM-PIAS, A modified procedure) for Hirschsprung disease have the advantage of low postoperative Hirschsprung associated enterocolitis. The controlled long-term follow-up studies evaluating Bowel Function Score (BFS) and Pediatric Quality of Life Inventory (PedsQoL, age <18 years) remain unclear.

**Methods:**

Between Jan 2006 and Jan 2016, 243 Patients underwent TRM-PIAS older than 4 years were included, while experienced redo surgery because of complication were excluded. Patients were compared with age- and gender-matched 244 healthy children each randomly selected from the 405 general population. The enrollee was investigated for questionnaires on BFS and PedsQoL.

**Results:**

One hundred and ninety-nine (81.9%) patients' representatives for the entire study population responded. The mean age of patients was 84.4 months (48–214 months). Compared with controls, patients reported impairment of hold back defecation, fecal soiling, and the urge to defecate (*P* < 0.05), and no significantly different in fecal accidents, constipation and social problems. With advancing age, the total BFS of HD patients improved, with a tendency close to the normal level beyond 10 years old. But, after grouped according to presence or absence of HAEC, the non-HAEC group experienced more dramatic improvement with age increasing.

**Conclusions:**

Compared with matched peers, significant impairment of fecal control prevails after TRM-PIAS in HD patients, but bowel function improve with age and recovery faster than conventional procedure. It should be emphasized that post-enterocolitis is a high-risk factor for delayed recovery.

## Introduction

Hirschsprung disease (HD) was first clinically described in 1,888. HD is characterized by an absence of enteric ganglia along a variable length of the intestine and can be classified as short-segment, long-segment, and total colonic aganglionosis (TCA) (1). This developmental disorder results in chronic functional obstruction.

Three standard procedures including the Swenson procedure, the Duhamel procedure and the Soave procedure have been described with varying functional outcomes (2). With advances in surgical techniques, data on long-term outcomes are slow to accumulate as surgeons continue to develop other types of operative treatments such as heart-shaped anastomosis, Z-shaped anastomosis, short-cuff Soave procedure and the Swenson-like approach transanal and laparoscopic techniques (3–5).

In 2014, our center described a modified approach in which patients with HD underwent a novel modification of transanal rectal mucosectomy and partial internal anal sphincterotomy (TRM-PIAS) (6). By reviewing 127 children with HD, this procedure has shown satisfactory short-term outcomes, especially with respect to HAEC and post-operative complications. However, the long-term bowel function and quality of life (QoL) after the TRM-PIAS operation in these patients have not been investigated. The present retrospective study aimed to define controlled outcomes for bowel function and QoL in HD patients after TRM-PIAS and compared to healthy subjects.

## Materials and methods

### Patients

**Figure 1:**
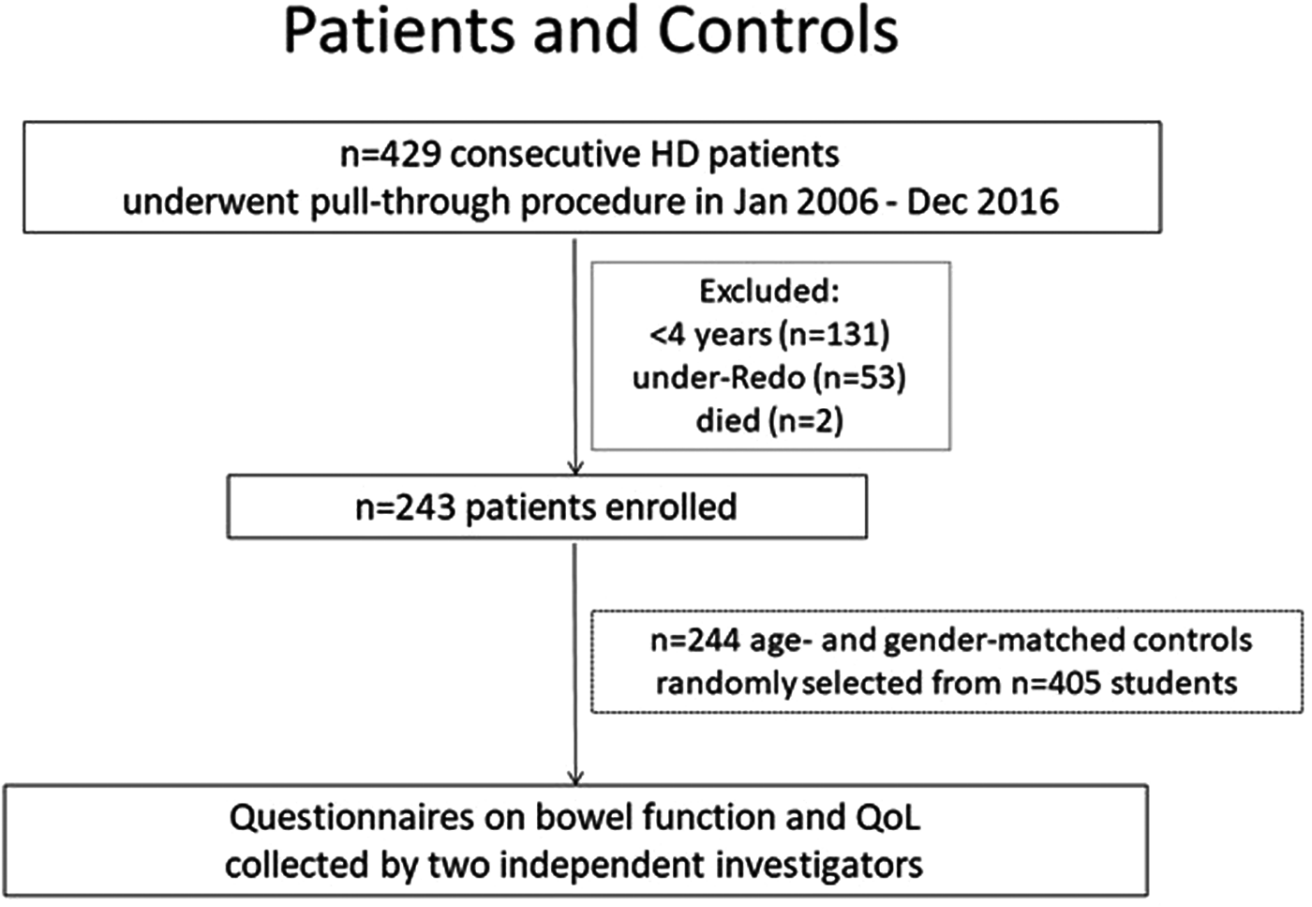
Flow chart.

Between January 2006 and December 2016, all patients diagnosed with HD at our center were identified as eligible for the study. From a total of 429 consecutive patients, children below the age of 4 years (*n* = 131) were excluded as these patients were not suitable for the long-term function measures and QoL questionnaires being utilized in this study. Fifty-three patients who received redo-operation due to complications after primary surgery in other centers (*n* = 38) and our center (*n* = 15) were also excluded. Furthermore, another 2 patients were excluded as 1 died from postoperative enteritis and the other died from congenital heart disease, neither of which were directly related to HD. Above all, in January 2019, a total of 243 patients were enrolled in this retrospective study. Two investigators distributed the electronic questionnaire to patients/parents, and reviewed the result on-line. When typing errors were suspected, the investigators would call the respondents by telephone and reconfirmed the filled result (Figure 1). Demographic and surgical data were obtained retrospectively from patient records.

### Controls

A total of 405 healthy children were randomly selected from three different schools and were asked to answer identical questionnaires which they received by mail. After being matched for age and sex, *n* = 244 controls aged 4–18 years were stratified for this study. None of the control patients had undergone any form of surgery or medical treatment in the last 3 months since being questioned.

### Enterocolitis

The guidelines for the diagnosis and management of HAEC have categorized clinical suspicion based on history, physical examination, and imaging studies (7). Persistent enterocolitis was defined based on continued or recurrent symptoms leading to 3 or more successive courses of medical treatment. This definition was used for HAEC in this study.

### Bowel function score

The Bowel Function Score (BFS) system, which was designed by R.J. Rintala in 1995, was developed for the assessment of bowel functional outcomes, with a maximum score of 20 (Supplementary Table S1). Individual items are scored 0–3, except for the frequency of defecation which is scored 1–2. A BFS ≥ 17 is interpreted as a good functional outcome.

### Quality of life questionnaires

The PedsQL™ questionnaires first developed by James W. Varni et al. in 1999, which have been previously validated by the World Health Organization, were used to assess QoL. It is a practical and validated modular instrument for measuring the pediatric health-related quality of life (HRQOL) in children aged 4–18 years (10). This scale is designed to measure the core dimensions of physical, emotional, social and school functioning (Supplementary Table S2).

### Operative technique

Transanal rectal mucosectomy and partial internal anal sphincterectomy (TRM-PIAS) is a modified Swenson procedure for HD which has been described by our center in 2014 (6). Briefly, TRM-PIAS was carried out circumferentially along the anorectal line. The anterior dissection was conducted between the rectal submucosal layer and the rectal muscular sleeve, while the posterior dissection was performed along the plane between the internal and external anal sphincters. The anus and rectum were serially dilated from size F8 to size F14 over 3–6 months. A physical examination and complications review was carried out 6 months post-operatively.

### Statistical analysis

Statistical analyses were performed using SPSS Version 21.0. Data are presented as frequencies or as mean with standard deviation. Categorical variables were compared using *χ*^2^ or Fisher exact test, and continuous variables using the Mann–Whitney *U* test. Results were considered statistically significant when the *P*-value was less than 0.05.

### Ethical approval

Ethics approval was obtained from the Ethics Committee of the Capital Institute of Pediatrics (DWLL2018009).

## Results

### Patient characteristics

One hundred and ninety-nine respondents (81.9%, 199/243) from parent-proxy were available. The basic clinical characteristics and surgical details of patients are shown in Table 1. The mean ages at operation was 17.3 months ranged from 0.6 months to 109.6 months. The mean follow-up period was 68.1 months, varying between 24.3 months and 119.9 months.

**Table 1:** Characteristics of the study population.

	Respondents (*N* = 199)	Nonrespondents (*N* = 44)	*P*
**Age (Month)**			
	84.4 + 30.7	91.6 + 32.1	0.042
**Sex (M: F)**			
Male	146 (73.4%)	36 (81.7%)	0.259
Female	53 (26.6%)	8 (18.3%)	
**Operative age (Month)**			
	16.3 + 22.5	18.7 + 26.6	0.38
**Level of aganglionosis**			
Rectosigmoid	162 (81.4%)	35 (79.5%)	0.832
Long-segment/Total colonic aganglionosis	37 (18.6%)	9 (20.5%)	
**Operation approach**			
TRM-PIAS	147 (73.9%)	32 (72.7%)	1
TRM-PIAS with Laparoscopy/Laparotomy	52 (26.1%)	12 (27.3%)	
**Associated syndrome**			
	7 (3.5%)	2 (4.5%)	0.235

Of those consecutive patients, 18.6% (*n* = 37) suffered serious HD type in had extended aganglionosis. Five patients presented associative syndromes including Down syndrome (*n* = 3) and Mowat-Wilson (*n* = 2).

### Drop-out analysis

To assess possible selection bias between respondents and non-respondents, a dropout analysis was performed. The length of the aganglionic segment, operation approach, and associated syndrome were comparable between respondents and non-respondents (*P* = n.s.). In drop-out analysis, the mean age of non-respondents is older than respondents (Table 1).

### Bowel function score

**Figure 2:**
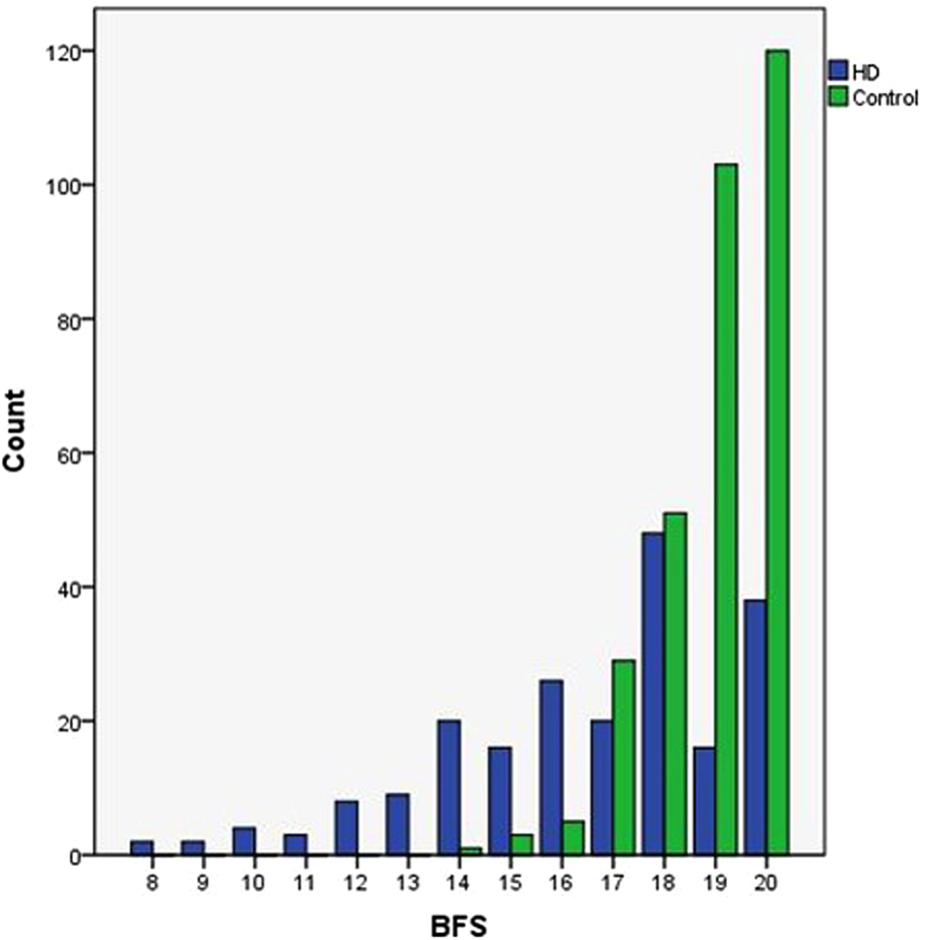
The respective medians (IQR) of bowel function score among patients with operated HD and controls. Distribution of individual scores is depicted.

The mean BFS was significantly lower in patients (17.99 ± 2.479) than in controls (19.2 ± 1.026, *P* < 0.001). The distribution of individual scores is shown in Figure 2. The bowel function was good (BFS ≥17) in 78.3% of patients and 97% of controls (*P* < 0.001), moderate (BFS = 12–16) in 18.6% of patients and 2.5% control, and poor (BFS < 12) in 3% of HD patients and none in controls.

A deeper analysis of the individual items assessing bowel function revealed that patients reported significantly more often the inability to hold back defecation, fecal soiling, and the urge to defecate (*P* < 0.05). In cross-section, holding back defecation, fecal soiling, and fecal accidents occurred in 6%, 22.1%, and 3% of patients, respectively. No patient had completely absented rectal sensation; completely normal recognition of the need to defecate was reported by 79.9% of patients (*n* = 159). The 2 females aged 5 with total colonic aganglionosis had total BFS scores of 9, and 3 males aged 5.7–6.9 years with rectosigmoid aganglionosis got 8 points of BFS. The percentages of patients and controls who reported any degree of respective functional impairment are shown in Table 2.

**Table 2:** In-depth analysis of individual items assessing bowel function.

	HD (*N* = 199)	Control (*N* = 244)	*P*
**Age (Year)**			
	7.03 + 2.595	7.37 + 2.955	0.289
**Feels the urge to defecate**			
Always	159 (79.9%)	219 (89.8%)	
Most of the time	22 (11.1%)	25 (10.2%)	
Uncertain	18 (9.0%)	0 (0%)	
Absent	0	0 (0%)	
Mean score + SD	2.71 + 0.624	2.90 + 0.304	<0.001
**Ability to hold back defecation**			
Always	153 (76.9%)	227 (93.0%)	
Problems less than once a week	34 (17.1%)	17 (7.0%)	
Weekly problems	8 (4.0%)	0 (0%)	
No voluntary control	4 (2.0%)	0 (0%)	
Mean score + SD	2.69 + 0.646	2.93 + 0.255	<0.001
**Fecal soiling**			
Never	68 (34.2%)	211 (86.5%)	
Staining less than once a week	87 (43.7%)	31 (12.7%)	
Frequent soiling, change of underwear required	35 (17.6%)	2 (0.8%)	
Daily soiling, protective aids required	9 (4.5%)	0 (0%)	
Mean score + SD	2.08 + 0.834	2.86 + 0.374	<0.001
**Fecal accidents**			
Never	182 (91.5%)	223 (91.4%)	
Less than once a week	11 (5.5%)	21 (8.6%)	
Weekly accidents, protective aids often required	3 (1.5%)	0 (0%)	
Daily, protective aids required day and night	3 (1.5%)	0 (0%)	
Mean score + SD	2.87 + 0.485	2.86 + 0.374	0.227
**Constipation**			
No constipation	183 (92.0%)	205 (84.0%)	
Manageable with diet	10 (5.0%)	37 (15.2%)	
Manageable with laxatives	5 (2.5%)	2 (0.8%)	
Manageable with enemas	1 (0.5%)	0 (0%)	
Mean score + SD	2.88 + 0.428	2.83 + 0.396	0.182
**Social problems**			
No social problems	182 (91.5%)	216 (88.5%)	
Sometimes (fouls odors)	13(6.5%)	28(11.5%)	
Problems causing restrictions in social life	4 (2.0%)	0 (0%)	
Major social/psychosocial problems	0 (0%)	0 (0%)	
Mean score + SD	2.89 + 0.368	2.89 + 0.319	0.778
**Frequency of defecation**			
Every other day-twice a day	174 (87.4%)	217 (88.9%)	
More often/ Less often	25 (12.6%)	27 (11.1%)	
Mean score + SD	1.87 + 0.332	1.89 + 0.314	0.627
**Total bowel function score**			
	17.99 + 2.479	19.20 + 1.026	<0.001

### Quality of life

The results of QoL questionnaires in patients and controls are shown in Table 3. Patients (*n* = 43) had worse scores than controls (*n* = 96), 86.5 + 15.30 vs. 91.8 + 8.65, *P* = 0.01) in the total PedsQL. In the individual items of PedsQL, patients had more frequent absences from school or daycare due to hospital visits than controls (*P* = 0.005).

**Table 3:** Comparison of quality-of-life questionnaire scores in patients and controls.

	HD Patient (*N* = 43)		Control (*N* = 96)		*P*
	Mean	SD	Mean	SD	
Physical score	90.77	15.06	94.17	11.02	0.137
Emotional score	85.81	17.59	90.63	12.94	0.073
Social score	90.00	15.92	95.05	8.41	0.016
School score	79.65	19.41	87.40	11.92	0.005
Total	86.56	15.30	91.81	8.65	0.011

### Predictors of bowel function

Seven risk factors including sex, time of surgery, aganglionic segment, operation approach, associated syndrome, increasing age and postoperative enterocolitis were as independent covariates in multivariate logistic regression analysis to assess predictors of BFS ≥ 17. Of these, increasing age was a significant predictor (OR 2.48, 95% CI, 1.02–6.02, *P* = 0.045 in 7–10 years old: OR 12.31, 95% CI, 1.48–102.05, *P* = 0.020 beyond 10 years old) of good functional outcome. Meanwhile patients who experienced ≥1 episode of postoperative enterocolitis were significantly more likely to have poor functional outcome (OR 10.44, 95% CI, 3.79–28.71, *P* < 0.001) (Table 4). Next, the mean bowel function score was analyzed with regard to subgroups of patients, and showed that there was no significant difference for any of risk factors other than age-stage and enterocolitis.

**Table 4:** Predictors of good bowel function determined from univariate analysis and multivariable analysis.

	no. of BFS ≥ 17	Univariable analysis		Multivariable analysis	
		OR (95% CI)	*P*	OR (95% CI)	*P*
Overall	156 (78.3%)	–	–		
Sex					
Male	115 (78.8%)	1.00 (reference)	–		
Female	41 (77.4%)	0.98 (0.79–1.20)	0.847		
Age at surgery					
<3 months	37 (77.1%)	1.00 (reference)	–		
3–12 months	70 (77.8%)	1.03 (0.54–1.97)	0.926		
>12 months	49 (80.3%)	1.17 (0.56–2.41)	0.814		
Level of aganglionosis					
Rectosigmoid	130 (80.2%)	1.00 (reference)	–		
Long-segment/Total colonic aganglionosis	26(70.3%)	0.89(0.74–1.08)	0.19		
Operation approach					
TRM-PIAS	119 (81.0%)	1.00 (reference)	–		
TRM-PIAS with Laparoscopy/Laparotomy	37(71.2%)	0.85(0.67–1.08)	0.170		
Associated syndrome					
YES	4 (57.1%)	1.00 (reference)	–		
NO	152(79.2%)	2.72(0.63–11.70)	0.173		
Age					
kindergarten (4–7 years old)	78(70.3%)	1.00 (reference)	–		
early elementary grades (7–10 years old)	51(85.0%)	1.98(1.02–3.86)	0.040	2.48 (1.02–6.02)	0.045
late elementary grades (>10 years old)	27(96.4%)	8.32(1.19–58.26)	0.003	12.31(1.48–102.05)	0.020
Enterocolitis					
YES	8 (34.8%)	1.00 (reference)	–		
NO	148(84.1%)	6.80(3.09–14.97)	<0.001	10.43 (3.79–28.71)	<0.001

### Effects of increasing age on functional outcomes

**Figure 3:**
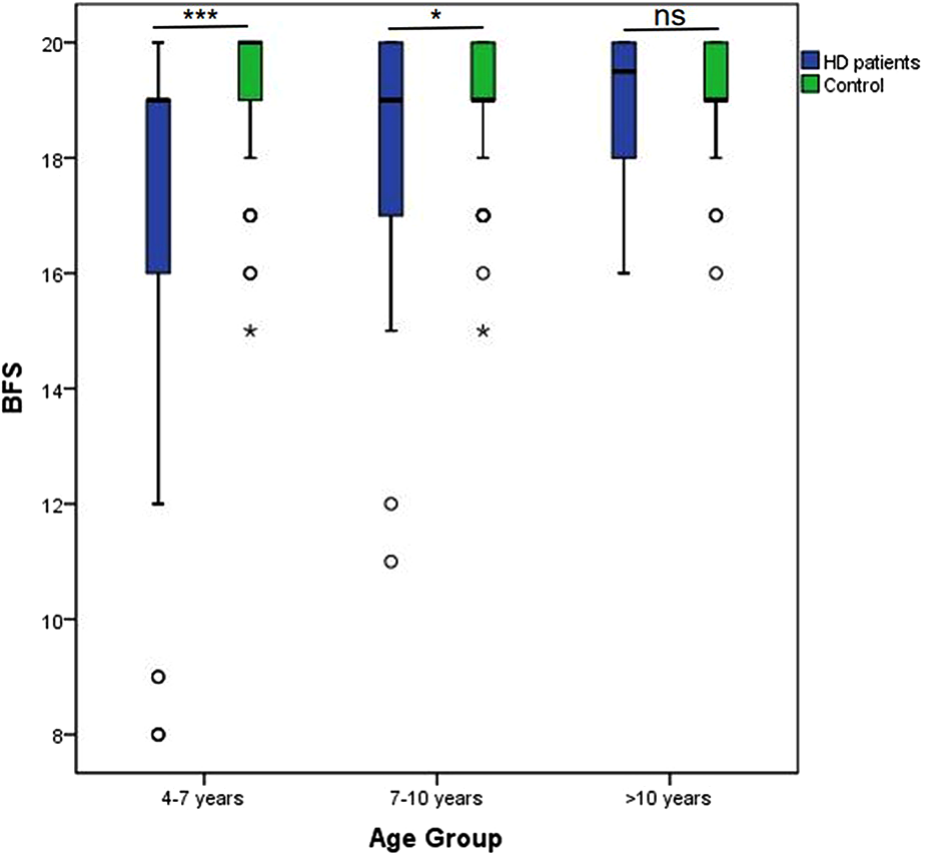
Effects of increasing Age on functional outcomes. With advancing age, total BFS of HD patients improved, with the tendency close to normal level.

With advancing age, the total BFS of HD patients improved, with a tendency close to the normal level. Among children in kindergarten (4–7 years old) and early elementary grades (7–10 years old), the BFS of HD patients was significantly lower than healthy children (17.47 ± 2.805 vs. 19.24 ± 0.991, *P* < 0.001 and 18.24 ± 2.079 vs. 19.11 ± 1.137, *P* = 0.019). At late elementary grades (>10 years old), the patients' bowel function could reach a similar level as their peers (19.06 ± 1.076 vs. 19.27 ± 0.939, *P* = 0.379) (Figure 3). Moreover, the rate of rectal sensation, fecal soiling, and total bowel function score differed significantly between these age groups (Supplementary Table S3 and Figure S1).

### Effects of enterocolitis on functional outcomes

Based on outpatient records for the first 12 months post-operation, 11.6% of the patients eligible for bowel function analysis (*n* = 23/199) had experienced early enterocolitis. The patients who had experienced persistent enterocolitis had significantly different proportions of aganglionic segments: 1.2% (*n* = 2/166) at short segment type, 12.1% (*n* = 4/33) at long segment type, and TCA (*P* < 0.001 between groups). Overall, a good bowel function outcome (BFS ≥ 17 points) was present in a lower proportion of patients with a history of enterocolitis than among enterocolitis-free patients (34.7% vs. 84.0%, *P* < 0.001) (Supplementary Table S4 and Figure S2).

**Figure 4:**
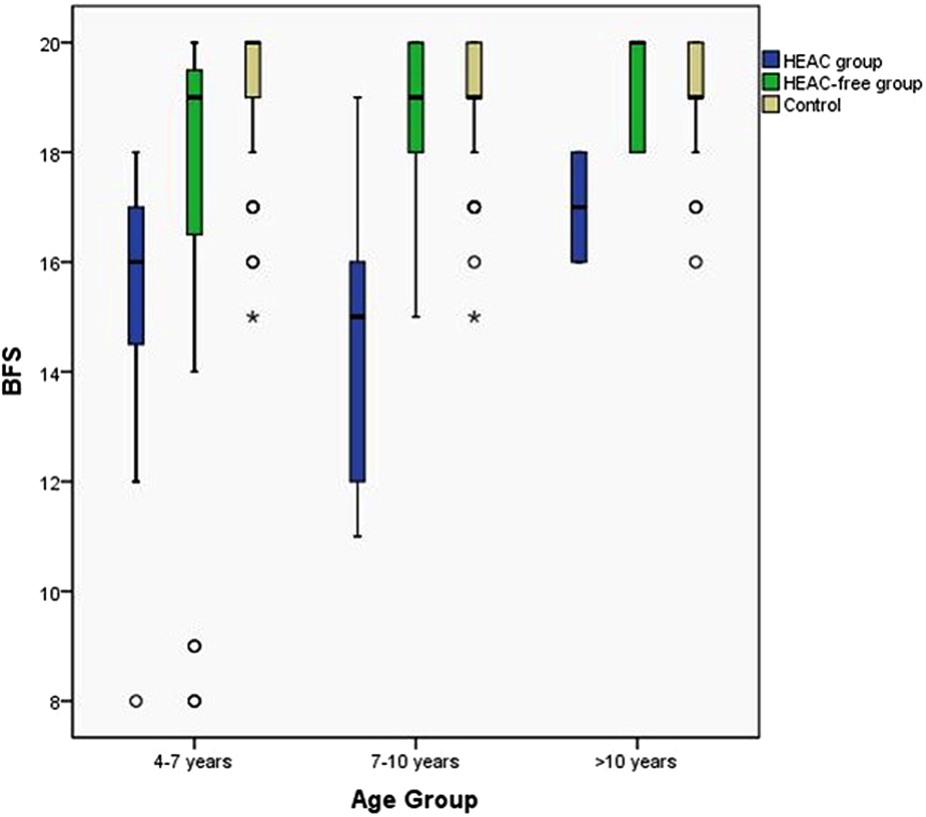
Effects of enterocolitis on functional outcomes with increasing age. The enterocolitis-free patients group bowel function outcomes reach healthy children level beyond 7 years old but the enterocolitis patients close to healthy children until 10 years old.

In combination with increasing age, both the non-HAEC group patients and HAEC group patients' bowel function are improved. Compared the BFS between these two groups, the non-HAEC group experienced more dramatic improvement (Figure 4). The enterocolitis-free patients were not significantly different from healthy children beyond 7 years old (18.93 ± 1.40 vs. 19.11 ± 1.14, *P* = 0.44), but the patients who had enterocolitis postoperatively still got a low score compared with healthy children (14.67 ± 2.875 vs. 19.11 ± 1.14, *P* = 0.0126). However, beyond the age of 10 years, no statistically significant association was noted between reported enterocolitis and bowel function impairment.

## Discussion

The operative management of HD remains the principle of removing the aganglionic bowel, pulling through the ganglionic bowel and preserving the anal canal and sphincter mechanism which can be done by an open laparoscopic-assisted transanal or total transanal procedure. The Soave procedure has been commonly used and preferred by surgeons worldwide because of the advantage of protecting the surrounding pelvic structures by an intramural plane of dissection (6, 11). However, The Levitt, MA team reported that from 2008 to 2012, thirty-six patients underwent reoperation for obstructive symptoms after an initial Soave pull-through for HD at their center.

Of patients who do poorly after their pull-through operations, a finite number will have mechanical issues which are specific to their individual surgical procedures. They suggested the Soave procedures leaves a long rectal aganglionic muscular sleeve surrounding the anastomosis which can lead to outlet obstruction or subsequent enterocolitis. Recognizing and dealing with complications in post-surgery, surgeon have developed a number of various novel surgical approaches and improved perioperative management have been developed during recent decades. There include transanal coloanal pull-through with a short muscular cuff (R J Rintala reported in 2003), long cuff dissection and short V-shaped partially resected cuff anastomosis (Li Yang reported in 2012), transanal endorectal stepwise gradient muscular cuff cutting pull-through method (Yuanmei Liu reported in 2018), transanal pull-through modified Swenson procedure without leaving a muscular cuff (Kazuki Yokota reported in 2018). Our center firstly introduced partial internal anal sphincterectomy was performed for Hirschsprung disease according to the methods described by JinShan Zhang et al. Previous reports have shown satisfied outcome, however, the long-term function outcome after operation in these patients have not been investigated.

We found significant bowel function imperfections after operated HD. Forty-three out of 199 patients had a BFS <17, taken as the lower limit of normal among the controls derived from the general population. Our findings are better than the results of a Duhamel procedure study demonstrating satisfactory functional outcomes (BFS ≥17) and absence of enterostomy or major revisional surgery in 47% of 49 patients using the same bowel function questionnaire (12).

Analysis of individual questions showed soiling was the most common problem, experienced by 65.8% of the patients. Similar findings have also been made in population-based follow-up studies of 68% of patients (13). Postoperative obstruction is another prevalent symptom ranging from aggressive laxative regimens to re-operation. Our study suggests that the incidence of constipation in HD patients is similar to matched controls at any age. This compares favorably with rates of up to 38% that have been reported in series after the Duhamel, Soave, and Swenson procedures (14–17).

Given the difficulty often encountered in establishing a definitive diagnosis, the reported incidence of HAEC varies widely. In our study, HAEC rate is 11.6% of postoperation HD patients, which is significantly lower than 28.3% and 37% reported from two critical cited literature in “Guidelines for the diagnosis and management of Hirschsprung-associated enterocolitis” by American Pediatric Surgical Association Hirschsprung Disease Interest Group, J Hagens have systematic reviewed 57 studies, including 9,744 preoperative and 8,568 postoperative patients, and reported the pooled prevalence for postoperative HAEC was in total 18.2%. Based on our clinical experience, postoperative HAEC is likely due to rectal muscle cuffs and internal sphincters without normal relaxation function, but we partially removed it, so the incidence of enteritis gradually decreases with the prolongation and dilation after temporary anastomotic oedema and scarring.

These variable predictors of outcome described above in our analysis demonstrate an improved outcome with increasing age. Our findings are consistent with 110 consecutive cases studied over 30 years in Japan in which the evacuation score improved chronologically and achieved satisfactory results at least 10 years after operation (18). In this study, we noted that partial resection of the internal sphincter did not significantly affect defecation function while reducing the recurrence rate of constipation. Previously, we thought that the internal sphincter played an important role in maintaining contractions, maintaining resting pressure, and avoiding incontinence. However, we found that partial internal sphincter resection not only effectively alleviates recurrence of constipation and enterocolitis caused by outlet obstruction after surgery; At the same time, it did not increase the occurrence of fecal soiling and incontinence. We consider that the function of the internal sphincter may be progressively compensated by the external sphincter, and that the muscular layer of the neorectal wall may also play a partial role as an internal sphincter. With the prolongation of time, the frequency of intestinal peristalsis gradually decreases, the water absorption capacity is enhanced, the new rectum also has a certain ability to store feces, and the child's awareness of defecation increases with age, and the long-term prognosis is more satisfactory.

As the proportion of patients who had experienced at least 1 episode of persistent enterocolitis did not increase in the analysis by age group, this suggests that the most difficult episodes may occur at a young age and cluster among certain patients. Interestingly, by focusing on cross-over analysis of increasing age and early HAEC, our result identifies the non-HAEC patients have a faster recovery process and favorable defection function at 7 years old. In the follow-up, we found that the prognosis of children with frequent enteritis is significantly worse than that of children without enteritis, and the possible reasons include: (1) the main cause of enteritis is outlet obstruction, and the recurrence of constipation caused by outlet obstruction are important factors affecting the prognosis. (2) Long-term chronic diarrhea caused by enteritis is more difficult to control stool, and it is more likely to lead to feces and incontinence. (3) Repeated hospital treatment and peculiar smell due to incontinence affect the normal social communication of children. (4) Malnutrition caused by repeated enteritis affects the physical development of the child.

## Conclusion

Compared with matched peers, significant impairment of fecal control prevails after transanal rectal mucosectomy and partial internal anal sphincterectomy pull-through in HD patients during childhood, but symptoms diminish with age. Meanwhile, we point out post-operation enterocolitis impair the recovery process.

## Data Availability

The original contributions presented in the study are included in the article/**Supplementary Material**, further inquiries can be directed to the corresponding author/s.
