# Supplementary material for: Long-term Bowel function and pediatric health-related quality of life after transanal rectal mucosectomy and partial internal anal sphincterectomy pull-through for Hirschsprung Disease
Source: Front Pediatr. 2023 Feb 14;11:1099606. doi: 10.3389/fped.2023.1099606 (PMC9971990; doi:10.3389/fped.2023.1099606)
Supplement: Supplementary file 1 [file Datasheet1.pdf]

Supplementary Table 1. The Bowel Function Score (BFS) system, designed by R.J. Rintala in 1995, for the assessment of bowel functional outcomes with a maximum score of 20. Individual items are scored 0-3, except for the frequency of defecation which is scored 1-2. A BFS>17 is interpreted as a good functional outcome.

**Bowel Function Score (BFS)**	**Score**
**Feels the urge to defecate**	
Always	3
Most of the time	2
Uncertain	1
Absent	0
**Ability to hold back defecation**	
Always	3
Problems less than once a week	2
Weekly problems	1
No voluntary control	0
**Fecal soiling**	
Never	3
Staining less than once a week	2
Frequent soiling, change of underwear required	1
Daily soiling, protective aids required	0
**Fecal accidents**	
Never	3
Less than once a week	2
Weekly accidents, protective aids often required	1
Daily, protective aids required day and night	0
**Constipation**	
No constipation	3
Manageable with diet	2
Manageable with laxatives	1
Manageable with enemas	0
**Social problems**	
No social problems	3
Sometimes (fouls odors)	2
Problems causing restrictions in social life	1
Major social/psychosocial problems	0
**Frequency of defecation**	
Every other day-twice a day	2
More often/ Less often	1

Supplementary Table 2. The PedsQL™ questionnaire was used to assess QoL. This questionnaire is a practical and validated modular instrument for measuring the pediatric health-related quality of life (HRQOL) in children aged 4 to 18 years. The scale utilized measures the core dimensions of physical, emotional, social and school functioning.

Ped QL(4-12y) Parent_report

	**Never**	**almost never**	**sometime**	**often**	**almost always**
**Physical functioning**	100	75	50	25	0
walking more than one block	100	75	50	25	0
running	100	75	50	25	0
participating in sports activity or exercise	100	75	50	25	0
lifting something heavy	100	75	50	25	0
taking a bath or shower by him or herself	100	75	50	25	0
doing chores around the house	100	75	50	25	0
having hurts or aches	100	75	50	25	0
low energy level	100	75	50	25	0
**Emotional functioning**	100	75	50	25	0
feeling afraid or scared	100	75	50	25	0
feeling sad or blue	100	75	50	25	0
feeling angry	100	75	50	25	0
trouble sleeping	100	75	50	25	0
worrying about what will happen to him or her	100	75	50	25	0
**Social functioning**	100	75	50	25	0
getting along with other children	100	75	50	25	0
other kids not wanting to be his or her friend	100	75	50	25	0
getting teased by other children	100	75	50	25	0
not able to do things that other children his or her age can do	100	75	50	25	0
keeping up when playing with other children	100	75	50	25	0
**School functioning**	100	75	50	25	0
paying attention in class	100	75	50	25	0
forgetting things	100	75	50	25	0
keeping up with schoolwork	100	75	50	25	0
missing school because of not feeding well	100	75	50	25	0
missing school to go to the doctor or hospital	100	75	50	25	0

Supplementary Table 3. Effects of age on various aspects of fecal control including urgency, rectal sensation, ability to hold back defecation, and fecal accidents, as well as constipation, social problems and total bowel function score.

	4-7years	7-10years	>10years	p
**Feels the urge to defecate**	72.0%	86.6%	96.4%	**0.020**
**Ability to hold back defecation**	73.8%	75.0%	92.8%	0.272
**Fecal soiling**	25.2%	43.3%	50.0%	**0.013**
**Accidents**	86.4%	96.6%	100.0%	0.164
**Constipation**	87.3%	96.6%	100.0%	0.117
**Social problems**	87.3%	96.6%	96.4%	0.214
**BFS**	70.2%	85.0%	96.4%	**0.004**

Supplementary Table 4. Effects of early enterocolitis on various aspects of fecal control including urgency, rectal sensation, ability to hold back defecation, and fecal accidents, as well as constipation, social problems and total bowel function score.

	non-HEAC Group	HEAC Group	P
**Feels the urge to defecate**	85.20%	39.10%	**<0.001**
**Ability to hold back defecation**	80.60%	47.80%	**0.002**
**Fecal soiling**	38.60%	0.00%	**<0.001**
**Fecal accidents**	93.10%	78.20%	0.115
**Constipation**	94.30%	73.90%	**0.001**
**Social problems**	93.10%	78.20%	**0.019**
**BFS**	84.00%	34.70%	**<0.001**

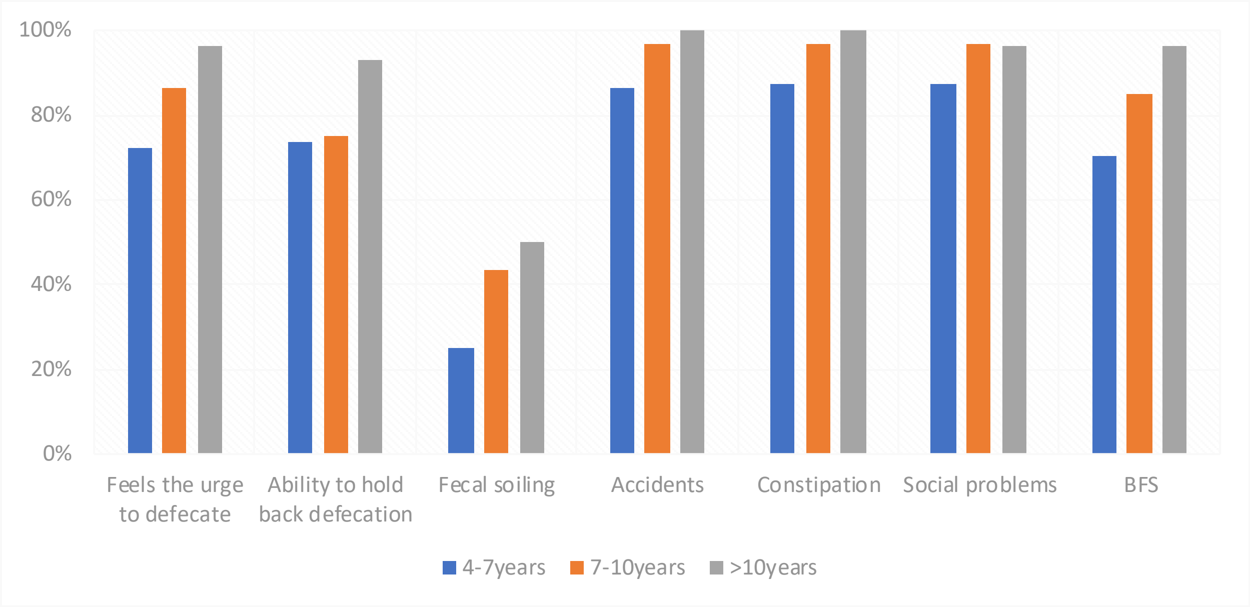

Supplementary Figure 1 Evolution of individual bowel function items in patients and matched controls of different ages. Percentage of subjects reporting great degree(score=3) of respective impairment.

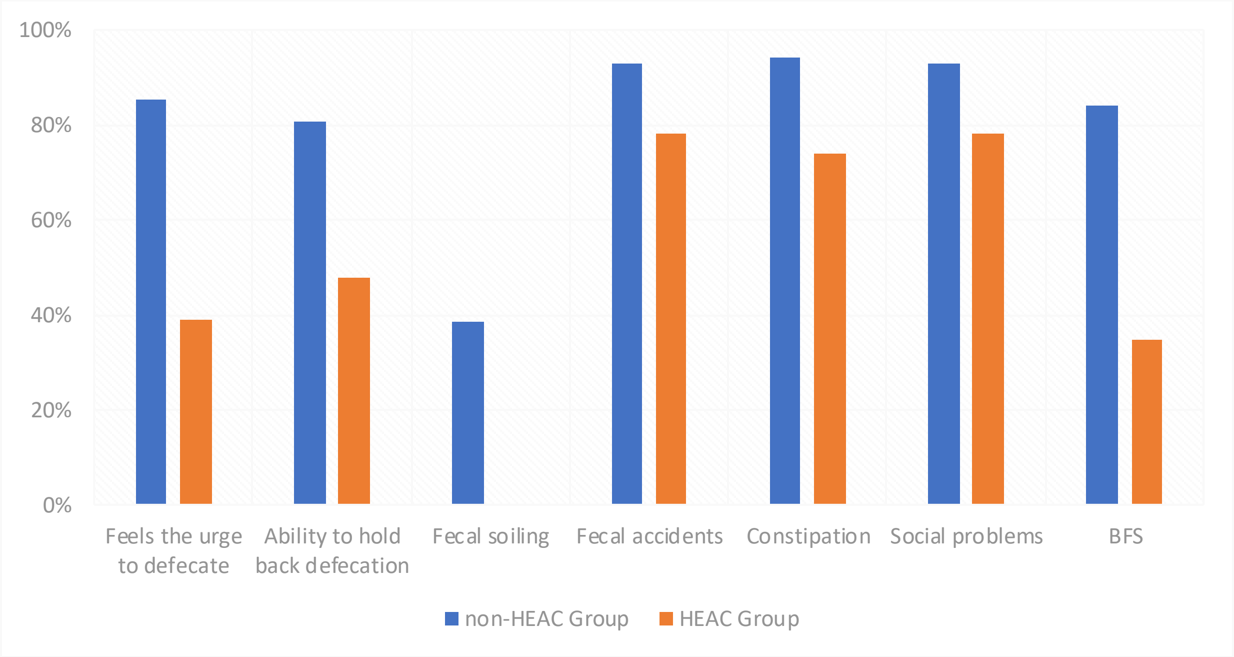

Supplementary Figure 2 Evolution of individual bowel function items in non-HAEC patients and HAEC patients. Percentage of subjects reporting great degree(score=3) of respective impairment.
